# Delineating the global plastic marine litter challenge: clarifying the misconceptions

**DOI:** 10.1007/s10661-020-8202-9

**Published:** 2020-04-04

**Authors:** John N. Hahladakis

**Affiliations:** Center for Sustainable Development, College of Arts and Sciences, Qatar University, P.O. Box: 2713, Doha, Qatar

**Keywords:** Plastics, Marine litter, Oceans, Plastic waste, Marine pollution, Plastic debris

## Abstract

Plastics, owing to their various beneficial properties (durability, flexibility and lightweight nature), are widely regarded as the workhorse material of our modern society. Being ubiquitously and increasingly present over the past 60 years, they provide various benefits to the global economy. However, inappropriate and/or uncontrolled disposal practices, poor waste management infrastructure, and application of insufficient recycling technologies, coupled with a lack of public awareness and incentives, have rendered plastic waste (PW) omnipresent, littering both the marine and the terrestrial environment with multifaceted impacts. The plastic marine litter issue has received much attention, especially in the past decade. There is a plethora of articles and reports released on an annual basis, as well as a lot of ongoing research, which render the issue either to be overexposured or misconstrued. In addition, there are several misinterpretations that surround the presence and environmental impact of plastics in the oceans and, consequently, human health, that require much more critical and scientific thinking. This short communication aims at unveiling any existing misconceptions and attempts to place this global challenge within its real magnitude, based either on scientific facts or nuances.

**Figure:**
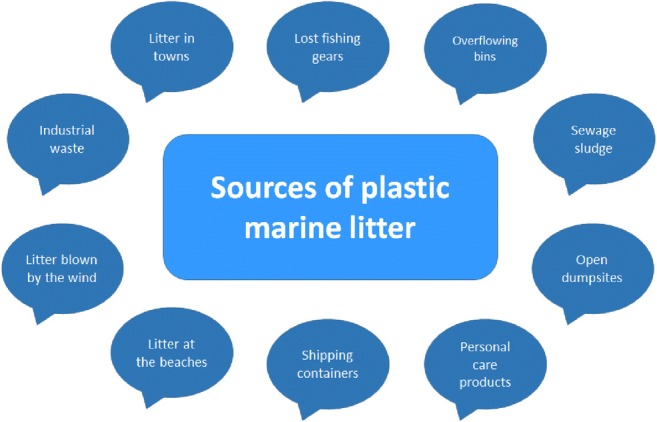
Graphical abstract

Graphical abstract

## Introduction

Under the circular economy (CE) perspective, we should retain resources in use perpetually, extract any value from them, plus recover and recycle products and materials at the end of their lifecycle. Plastics, with their lightweight, durable, and versatile nature, could majorly attribute on a more sustainable and resource efficient future. Being applicable to many sectors such as packaging, electrical and electronic equipment (EEE), construction, and automotive, they could save energy and resources. In addition, plastic packaging can help reduce product losses (e.g., food waste).

Conventional plastics are—mostly—made of thermoplastic resins and can be generally categorized into seven classes: the polyethylene terephthalate (PET) (known as type 1); high-density polyethylene (HDPE) (known as type 2); polyvinyl chloride (PVC) (known as type 3); low-density polyethylene (LDPE) (known as type 4); polypropylene (PP) (known as type 5); polystyrene (PS) (known as type 6); and others (known as type 7). The latter category refers to multilayer polymer formations, not collected for recycling (Hahladakis and Iacovidou 2018).

Plastics are nowadays ubiquitously present, with an increased production that reached approx. 322 Mt (in 2015), with a projection of doubling this amount by 2035 (Ellen MacArthur Foundation 2016; Geyer et al. 2017; PlasticsEurope 2016). The European Commission (EC) has recently introduced a European Strategy for Plastics (European Commission 2018), after having already identified and set any action on this type of product as a priority in the 2015 CE Action Plan (European Commission 2015); facts that solidify their significance and highlights the need for further future research and investigation (European Commission 2016, 2018).

**Fig. 1:**
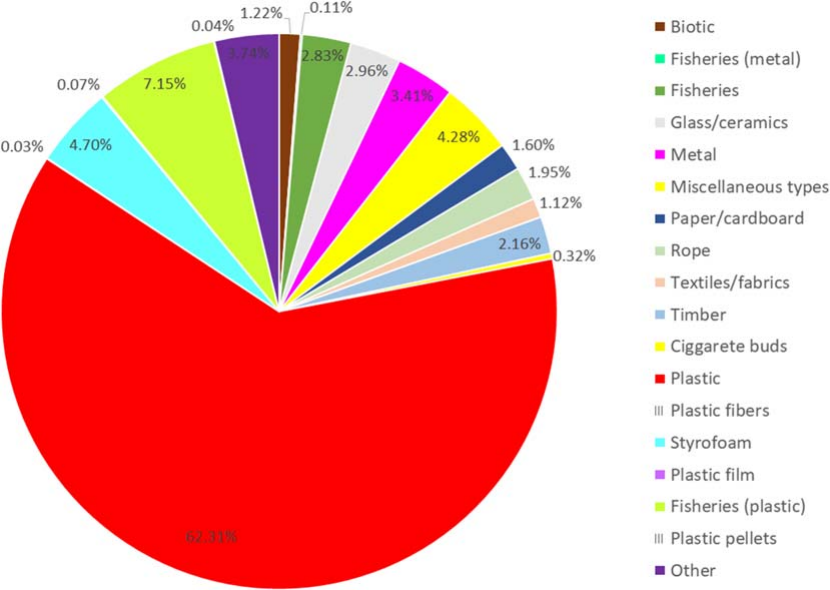
Global composition estimate of marine litter. The percentages of the various types of litter shown in the graph were calculated as the weighted means of all studies under consideration, regardless of units (565 publications, 3982 locations). (Redrawn from source: https://litterbase.awi.de/litter_graph)

However, their value chain is still treated with the archetypically linear mode of take-make-dispose. In addition to that, inappropriate and/or uncontrolled disposal practices, poor waste management infrastructure, and application of insufficient recycling technologies, coupled with a lack of public awareness and incentives, have rendered plastic waste (PW) the major component of marine debris. According to Awi-Litterbase, a continuously updating database on global marine litter, plastic affiliated debris accounts for approx. 70% (see Fig. 1) (Tekman et al. 2019). While plastics litter the terrestrial environment, too, presenting multifaceted impacts, they will inevitably reach the oceans, being our planet’s ultimate sink (Jambeck et al. 2015, 2017). It is only during the past few years, together with the launch of the CE concept, in 2010, that the drawbacks of this way of dealing with plastics have been, at last, clearly realized (Ellen MacArthur Foundation 2010, 2012, 2013, 2014).

Plastics can, also, be classified into three groups, based on their particle size. All plastic materials > 5 mm fall under the category mostly known as “macroplastics” (Axelsson and van Sebille 2017). Nonetheless, due to various conditions and environments (e.g., weather, UV light, seawater, etc.), plastics can potentially be degraded and dissociated (fragmented) into smaller pieces, 50 μm–5 mm, called microplastics (MPs) (Andrady 2011; Kalogerakis et al. 2017; Wang et al. 2016). Finally, the nanometer-sized plastic particles, usually defined in < 100 nm of size, constitute the “nanoplastics” group (Koelmans et al. 2015).

**Fig. 2:**
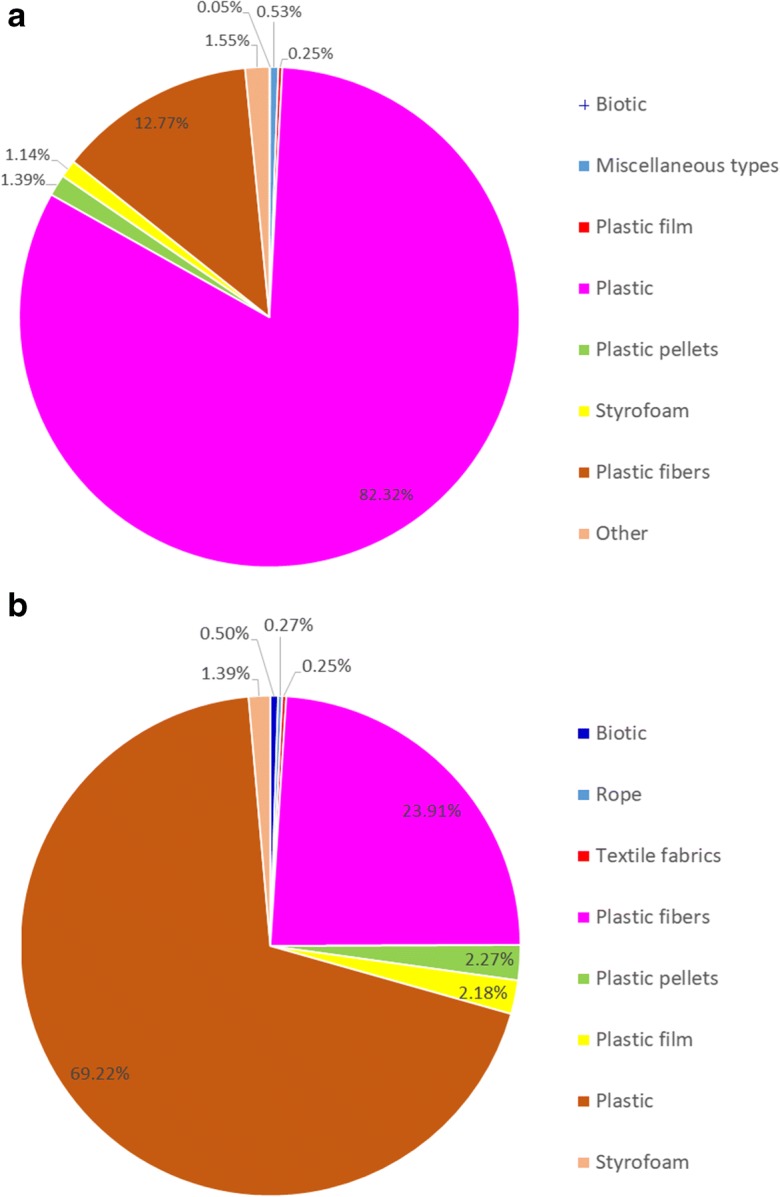
Micro-litter composition found **a** in the water column (53 publications, 554 locations) and **b** in the benthos (82 publications, 689 locations). (Redrawn from source: https://litterbase.awi.de/litter_graph)

It is, in fact, the latter two categories that are considered the most potentially harmful, both to humans as well as to other living organisms, especially when present under aquatic conditions, due to several reasons: (a) after, e.g., entering the marine environment, a large part of it is out of sight because it lies below the surface (see Fig. 2), (b) they can easily be ingested or entangled by various species (Browne et al. 2008; Steer et al. 2017; Teuten et al. 2009), and (c) embedded chemical substances (additives) are more readily released during the degradation process of these fine particles rather than the larger ones. Furthermore, these tiny pieces that can easily accumulate persistent organic pollutants (POPs) and other substances of concern (SoC) (Chen et al, 2019; Hahladakis et al. 2018; Koelmans et al. 2013) serve as a pathway to food chain.

So, naturally, a series of questions arise; how serious and emerging is the problem of plastic marine litter on our planet? What measures can be taken—both upstream and downstream—to reduce the amount of plastic waste that end up as marine litter and mitigate their effects? Are these effects irreversible?

Via this short communication, the author aims to address the aforementioned questions, by listing a series of statements that have already been reported in literature, attempting to clarify any hidden misconceptions, and revealing the true magnitude of the global plastic marine litter issue based on scientific facts and nuances.

## Uncertainties or facts?

### Plastics vs fishes

It was recently estimated and reported that if the linear “take, make, use, and dispose” model of economy continues to prevail and society fails to implement a successful CE model, then by 2050, there will be more plastics, by weight, in the oceans than fish (Ellen MacArthur Foundation, 2016). Considering the several assumptions and uncertainties entailed in the methods used to estimate both the fish population, as well as predict the amount plastics that will be present in the oceans by 2050, it is almost impossible to verify and support that kind of statement (Galloway and Lewis 2017; Homak 2016; Watson et al. 2017). Regardless of the obvious inaccuracies entailed in this statement, it was widely quoted and regurgitated; nonetheless, the figures presented therein represent mostly an attempt to underpin the urgency of the issue, rather than provide a verifiable scientific estimate.

### 10 rivers do 90% of the “damage”!

**Fig. 3:**
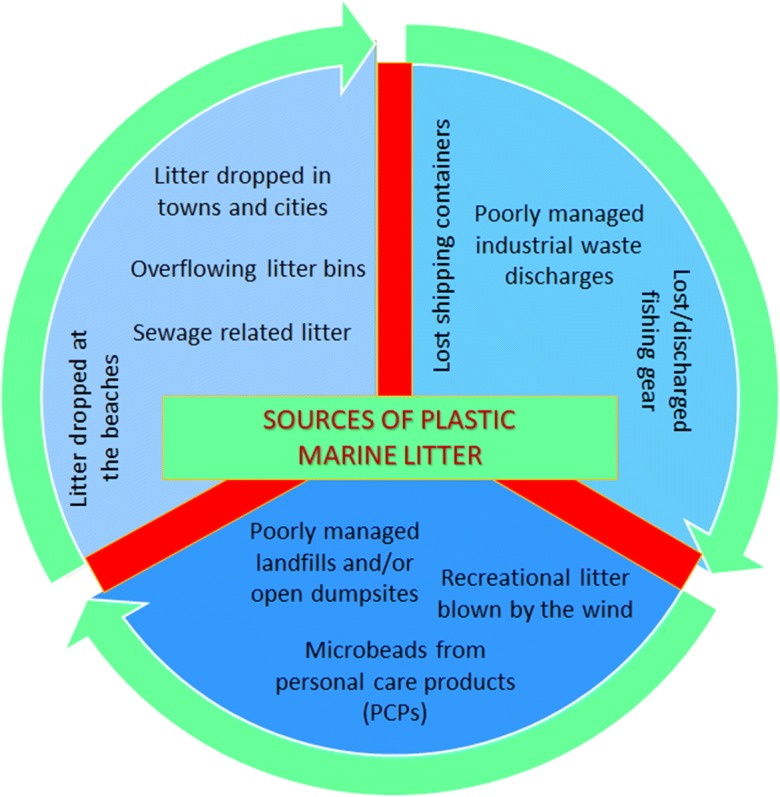
Sources of marine (plastic) litter

There are various pathways and origins for PW that ends up into the oceans. A nice infographic, sourced by Cleancoasts Org. and shown in Fig. 3, depicts the major sources of marine litter. It can either be directly disposed, wind-blown, dumped from cruise ships, and/or tangled in fishing nets. Rivers are also considered one of the major plastic carriers. The most recent estimate of the total amount of plastics entering the oceans is approx. 8 Mt per year, out of which ca. 80% is attributed to land-based sources (Jambeck et al. 2015). Other studies report that an approx. 6.2 Mt of macroplastics and 3.0 Mt of MPs were lost to the environment in 2015, identifying as a major loss source of macroplastics (4.1 Mt) mismanaged municipal solid waste (Ryberg et al. 2019). However, due to a lot of assumptions and inaccuracies, the aforementioned figures cannot be verified.

In the recent study of Schmidt et al. (2017), a calculation of the approx. amount of PW transported by 57 individual river systems worldwide was attempted. The results showed that 10 rivers (Indus, Ganges, Amur, Hai he, Yellow, Mekong, Pearl, Yangtze, Nile, and Niger) could be held accountable for approx. 88–95% of it (Schmidt et al. 2017). This does not, of course, imply that ca. 90% of the total PW existing in the oceans derives from these 10 rivers. Despite the uncertainty entailed in this estimate (within a range of anywhere between 0.4 and 4 Mt per year), it is indicative of the contribution of rivers in the global plastic marine litter situation. The results of this study can also help in targeting the regions that lack a proper waste management infrastructure and where relative actions are needed.

Consequently, even though river-borne plastics constitute a major source of marine litter, respective scientifically supportive data is not yet available (UNEP 2016a); hence, other sources of marine pollution should not be ignored and/or underestimated. The majority of plastics that burden our oceans is affected by societal attitudes, both on land and at sea, as well as from the dynamics of the producing-generating waste systems (Horton et al. 2017). According to UNEP and GRID-Arendal (2016), all sectors and players contribute to this pollution through various ways, e.g., open dumpsites, uncontrolled landfills, poorly disposed waste on land, plastic debris from fishing activities, ship merchant leakages, cruise ships, etc. (UNEP and GRID-Arendal 2016).

As research continues, the major sources of plastic pollution in the oceans will soon be verified and classified according to their quantifiable contribution. In the meantime, action by all individuals is deemed necessary, while governmental entities should take immediate measures to tackle this global challenge.

### Plastic is our ocean’s “number one” threat!

During the past 20 years, plastic pollution has been presented as a significant threat to our natural marine environment.

According to estimates by Eriksen et al. (2016), 5.25 × 10^12^ plastic pieces float in our oceans; a number equivalent to approx. 700 pieces per living human being on Earth, given an earth’s population estimate of 7.5 billion people (Eriksen et al. 2014). These plastics should weigh more than 225 kt.

In another study, calculations were found anywhere between 15 and 51 trillion pieces, the majority of which were micro and nanoparticles (Worm et al. 2017). Naturally, debris of any size is considered harmful to marine biota, since marine animals could mistakenly eat it or become entangled to it (UNEP 2016a).

Nonetheless, although marine plastic pollution is considered among the most prevalent threats, an equally important issue is how it affects and/or alligates other benthic compounds or stresses the oceanic balance. These challenges and/or threats may include overfishing, climate change, acidification, ocean warming, and habitat destruction (Halpern et al. 2008). This combination of anthropogenic-derived impacts, coupled with our delayed and/or inexistent willingness to address them, may constitute the real threat to oceans, and while we may assume that it is too late, it is actually not!

### The smaller it is… the harmer it gets! The case of personal care products (PCPs)

There is an increasing concern on the effects marine MPs could potentially have on humans, including the leaching of various SoC embedded in them, and their ability to enter into the food chain, thereby affecting human health. In fact, there have been several efforts to have them classified as POPs owning to their pervasive and persistent nature (Hurley et al. 2018). But how harmful do they really are?

It seems that the marine food chain is full of plastics, mostly found in the stomachs of fishes in the Northwest Atlantic (Wieczorek et al. 2018), in the English Channel (Lusher et al. 2013), in endangered sea turtles (Caron et al. 2018), in the Bluefin tuna nearby the coast of Lebanon (Trtrian 2018), and in the stomach of a dead whale nearby the coast of Norway (The Associated Press, 2017). It is speculated that when ingested, MPs provide a sense of completeness in the fishes’ stomachs, thereby leading them to weight loss and potential death due to starvation. In addition, when smaller fishes are eaten by predators, plastics move up the food chain. Various chemicals could potentially be adsorbed to MPs, thereby increasing accumulation of toxins in larger predatory fish that will potentially end up on our plates. Such types of toxins may penetrate cell membranes, thereby increasing the exposure to SoC.

On the other hand, it has been reported that molecules with a molecular weight exceeding 1000 g/mol do not cross the colon-blood barrier and, thus, do not pose direct toxicological effects. For degraded polyethylene, this would equal an oligomer consisted of 36 monomers which corresponds to an approx. volume of 4.5 nm^3^. Such a size would exclude all the MP’s, as well as a large fraction of the nanoplastics. These tiny fragments are known as MOSH (mineral oils of saturated hydrocarbons) and POSH (polymeric oligomers of saturated hydrocarbons). However, there is still substantial toxicological debate on this matter (Koster et al. 2020; van de Ven et al. 2017).

In the case of PCPs, these include several MPs in the form of abrasive microbeads, mostly encountered in face cosmetics, or plastic granules mostly used in manufacturing (Mepex 2014). These particles will, eventually, end up in wastewater systems; however, depending on the efficacy of these systems, it is the oceans that could, unfortunately, be their final recipient (UNEP 2016b). In addition, the nanoparticles contained in sunscreens can wash off people’s skin during swimming or bathing. While there are numerous MPs in a single PCP, it has been estimated that only a limited amount of 4.6–9.5 thousands of microbeads can be released, with each application of a skin exfoliant (Napper et al. 2015). This number is considered relatively small, compared to other primary or secondary sources of MPs in the environment, with regard to tonnage (UNEP 2016a). So, while banning the use of cosmetic microbeads could restrict the amount of MPs entering the marine environment, it is not a panacea! Nonetheless, this example of microbeads could potentially serve as a useful illustration to raise public awareness about the extent of the marine litter challenge.

Assessing any human health risks related to plastic marine litter is a complex issue and a lot more to discover and investigate about its potential effects to human health (UNEP and GRID-Arendal 2016). In addition, as reported in UNEP (2016a), “the uptake of plastic-associated chemicals in humans due to inadvertent ingestion of MPs in seafood appears likely to be no more significant than other human exposure pathways of these chemicals” (UNEP 2016a).

So, despite the growing concern about the micro- and nanoplastics effects on humans, and while we keep on discovering new insights on this matter, further work is required to establish their effects on our health. So far, there are no scientific evidence that they are directly harmful to human health.

### Plastics…an “island” of litter in our oceans!

Despite the fact that plastic debris prefers to accumulate in specific areas in the oceans, there are no visible layers of plastic seen, e.g., from airplanes (Lebreton et al. 2018). This can possibly be attributed to the abundant presence of small or fragmented particles that cannot easily be seen or even stay afloat. However, the public is mostly bombarded with pictures of seas full of plastic bottles, bags, toys, and other large items. In these patches, the number of pieces has been reported to exceed 200,000 particles/km^2^, which equals to less than one microplastic particle/m^2^ (UNEP 2016a). Although larger pieces do occur, in general, the particles are widely dispersed and, given the dynamics of the marine ecosystem, it is extremely difficult to estimate their size (NOAA 2018).

There has been an increasingly concern with regard to the formation of a plastic patch in an area of the Barents Sea (Bergmann et al. 2016; Cózar et al. 2017); nonetheless, despite the potential effects in fishes, marine mammals, and seabirds (Hallanger and Gabrielsen 2018), densities found there have been reported to be slightly higher the Antarctica ones, and much lower than those from temperate waters (Bergmann et al. 2016).

However, regardless of the exact size, mass, and location of PW, anthropogenic-derived debris should be removed from the oceans.

### Plastics last forever!

It has recently been estimated that it will take more than 450 years for a plastic bottle to completely degrade (KABC 2018). However, plastic bottles were not widely used until the 1960s; so, it would be just a mere hypothesis for one to determine their exact lifetime, when present in aquatic environments.

Plastics disintegration can be caused by solar UV radiation and is most intensive in beaches, shorelines, and the water surface. However, their degradation rate is a multifactorial-dependent phenomenon; composition, temperature, wave abrasion, etc. are a few parameters to mention (Andrady 2015). The majority of the new generation manufactured plastics are considered extremely durable, able to persist for centuries (UNEP and GRID-Arendal 2016). Furthermore, it has not been evidenced whether a full degradation will ever occur (O’Brine and Thompson 2010). The larger particles will eventually fragment into smaller which, in turn, will take even longer to degrade; plus, the research on nanoplastics is still at an infant stage (Koelmans et al. 2015; Mattsson et al. 2015).

So, the “450 years” is not a verifiable lifetime duration. The only thing we can tell, so far, is that plastics take extremely long to break down; hence, preventing them from entering the marine environment is considered an urgent necessity.

## Potential solutions to the global plastic marine litter challenge

### Begin with what can be seen!

Collecting the floating particles might seem like a tempting and working solution; however, there are other more efficient alternatives, i.e., preventing plastics from entering the aquatic environments in the first place. The majority of marine plastics tend to be mostly found under the water surface, thereby rendering any surface clean-up attempt ineffective. In addition, collecting floating particles could result in the implementation of costly cleaning technologies which could negatively affect marine life and biodiversity (Thaler 2015). This is affiliated to the fact that marine biota could get used to clean-ups and/or get exposed to greater amounts of plastic (Thaler 2015). In addition, this type of surface cleaning equipment, which most of the time involves also the application of biofouling organisms, could cause further complications (Martini 2014). Besides, cleaning a coastline/shoreline could be much easier and efficient, rather than spending huge capitals for floating plastic collection equipment (Openchannels, 2015). Thinking locally and targeting to ecologically sensitive areas (e.g., touristic, fishing, etc.) could bring much better and immediate results, both for the well-being of local habitats, as well as for our oceans (in the long run). Such actions raise awareness about the plastic marine litter problem, can boost individual and local action, and can incentivize public to ask from producers and governments even more radical solutions (NOAA 2016).

### To go or not to go…“bio”?

Biodegradable and bio-based plastics that could disintegrate naturally without causing any environmental harm may appear as a perfect solution to the marine litter challenge; however, the reality is not that simple.

In a UNEP report on biodegradable plastics, it was reported that labelling plastic material, components, and products (MCPs) as “biodegradable” will not result in decreasing either the quantity or the risk plastics pose on the marine environment (UNEP 2015). The same report also noted that achieving a complete biodegradation of these types of plastics usually requires conditions that are not commonly found in the aquatic environments (UNEP 2015); so, they may even contribute to the existing problem. Although their current production is quite limited (representing about 1% of the 335 Mt of the annually global plastic production) (PlasticsEurope, 2017), there is evidence that public perceptions on the biodegradability of an item can influence their littering behavior; i.e., a biodegradable marked bag might lead them to appropriately dispose it (UNEP 2015).

The main issue is our attitude towards consuming plastics (especially single-use and disposable). Bioplastics or any other technological innovations could misguide us from adopting a new consuming and disposing behavior; that is where our true goal should be.

### The real solutions always lie within us!

Incentivizing the public into individual actions would be the ideal solution to address the global plastic marine litter issue. Changing our own mentality on waste disposing attitude can make a difference. In the long run, it will help reduce the total amount of PW that are inappropriately disposed and, ultimately those that enter the oceans. Worldwide examples of inspirational actions include the “Clean up Kenya,” the “Bye-Bye Plastic Bag,” and the “Last Straw.” In addition, San Francisco has banned plastic bags and bottles (Levin 2017). It is also noteworthy that the Indian state of Maharashtra and the EU are banning single-use plastics.

Keeping in mind all the aforementioned locally implemented program initiatives, each individual can contribute to tackling marine litter by adopting the below indicated actions:

- *Become an educated consumer*: disposing the waste in an appropriate and responsible manner will help reduce the amount that ends up in the oceans. Furthermore, selecting the same reusable items on a daily basis, e.g., a water-bottle, cosmetics that do not contain MPs, a shopping bag, a coffee cup, etc. is a waste behavioral changing habit.
- *Sorting and recycling*: recycling plastic leads to less virgin material produced and, thereby, less entering the marine environment. Despite how simple that may seem, there is still room for improvement in order to achieve recycling in a proper manner.
- *Support and implement direct action*: participating in local recycling and international campaigns will help us change our overall mentality towards production, use, and disposal of plastics.
- *Awakening governmental entities*: contacting the right people and informing them that this global issue is highly important to you. Persuade them to imitate and reproduce any of the aforementioned initiatives of Kenya, San Francisco, Bali, and many other places around the world by introducing specific legislations and policies.

## Conclusions

Plastic production, consumption, recovery, and recycle are a nexus affected by a web of different facets, occurring at different parts of the supply chain. Therefore, a multidimensional appraisal of the system as a whole, as well as several changes/interventions are needed to be carried out in order to prevent leakages to the environment and for sustainable developments to occur (Iacovidou et al. 2019).

Governments should cooperate not only on local but also on global level to regulate the main origins and sources of MPs. Taking into consideration that this issue is a relatively new and of increasing concern, more resources should be allocated to further research on the long-term effects and consequences that plastics, and additives contained in them, have on living organisms (Hahladakis et al. 2018; Oehlmann et al., 2009). Filling this knowledge gap could potentially contribute to the lack of certain regulations, regarding, e.g., the prevention or limitation in the use of bisphenol A (BPA).

Although dumping of PW is forbidden by the International Convention for the Prevention of Pollution from Ships (MARPOL) Annex V, many people are unaware or tend to ignore this. Several campaigns organized by relevant policy makers, marine businessmen, industries, and stakeholders should take place, so as further light is shed on the urgency of this international matter. Large multinational organizations, such as the United Nations Environment Programme (UNEP) and the International Maritime Organisation (IMO), should also contribute and organize campaigns, on their own, on a global scale.

Plastic industries should be responsible for the end-of-life of their products, using, as possible, biodegradable material that will be more easily degraded by microorganisms (such as bacteria and fungi), thereby reducing even more the lifetime of these bioplastics, when/if entering the marine environment (Gregory and Andrady 2003).

Finally, incentivizing and educating the public on the seriousness of the situation caused by plastic litter are considered an absolute necessity in stepping towards shifting people’s behavior with regard to plastic consumption, use, and disposal habits. It is a priority issue that should be placed on the top of the international political agenda.

## Abbreviations

approx.: Approximately
ca.: Circa (Latin term for “approximately” or “about”)
CE: Circular economy
EC: European Commission
EU: European Union
EEE: Electrical and electronic equipment
HDPE: High-density polyethylene
LDPE: Low-density polyethylene
MCPs: Materials, components, and products
MPs: Microplastics
PCR: Personal care products
PET: Polyethylene terephthalate
POPs: Persistent organic pollutants
PP: Polypropylene
PS: Polystyrene
PVC: Polyvinyl chloride
PW: Plastic waste
SoC: Substances of concern
UN: United Nations
